# Effects of Different Concentrations of Inhaled Oxygen on Lung Collapse and Oxidative Stress in Patients With One Lung Ventilation, a Single‐Center Prospective, Double‐Blinded, Randomized Controlled Trial

**DOI:** 10.1002/hsr2.71600

**Published:** 2025-12-02

**Authors:** Dizhou Zhao, Chujun Wu, Haoshuai Zhu, Jun Lin, Wangzhi Zhang, Jieyu Fang

**Affiliations:** ^1^ Department of Anesthesiology ShenZhen People's Hospital (The Second Clinical Medical College, Jinan University; The First Affiliated Hospital, Southern University of Science and Technology) Guangdong Shenzhen China; ^2^ Department of Anesthesia, the First Affiliated Hospital Sun yat‐sen University, Guangzhou China; ^3^ Department of thoracic surgery, the First Affiliated Hospital Sun yat‐sen University Guangzhou China; ^4^ Department of Anesthesia Guangzhou Women and Children's Medical Center, Guangzhou Medical University Guangzhou China

**Keywords:** lung collapse, one lung ventilation, oxidative stress, oxygenation level, thoracoscopic surgery

## Abstract

**Background:**

During thoracoscopic surgery, the anestheologist assure an adequate oxygen supply to the body, but at the same time, to improve the surgical field by completely collapsing the lung on the surgical side. However, incomplete lung collapse remains problematic. Increasing the inhaled oxygen concentration might accelerate gas dispersion from the alveoli, but continuous high oxygen may aggravate oxidative stress. The selection of inhaled oxygen concentration during OLV is still controversial. This study intends to explore this issue from the perspective of collapse effect and the degree of oxidative stress.

**Methods:**

This study included patients aged 18–65 undergoing thoracoscopic lobectomy, divided into two groups: one receiving 70% oxygen (70% group) and the other 100% oxygen (100% group) during surgery. Lung collapse was evaluated upon camera entry into the chest cavity, and blood samples were collected preoperatively, 30 min after OLV, and on the second postoperative day.

**Results:**

Both groups were similar in baseline characteristics. The 100% oxygen group showed significantly greater declines in lung volume and elasticity scores at 5 and 10 min post‐thoracic entry compared to the 70% group (*p* < 0.05). Oxygenation was significantly lower in the 70% group post‐OLV (*p* < 0.05) during surgery, and oxidative stress markers were higher in the 100% group during surgery (*p* < 0.05). By the second postoperative day, oxidative stress markers increased in both groups with no significant difference. There was no significant difference in safety outcomes between the two groups of patients.

**Conclusion:**

High/pure inhaled oxygen may improve the lung collapse speed and provide a better field of vision for thoracic surgery. Although pure oxygen ventilation aggravated short‐term oxidative stress, the effect was insignificant on the second postoperative day.

## 1 Introduction

The two‐lung separation technique is a critical procedure that partitions the airway above the tracheal carina or bronchus, enabling the lungs to be ventilated independently. This is achieved by the anesthesiologist, who skillfully inserts either a bronchial blocker or a double‐lumen tube (DLT) through the mouth and into the trachea. In the context of thoracoscopic surgery, this method of lung separation facilitates ventilation of one lung only, while the anesthesiologist simultaneously removes the gas from the operative side. This evacuation of gas is essential for creating a clear and unobstructed surgical field, which is crucial for the precision and safety of the surgical procedure [1, 2].

Partial lung collapse, which can be attributed to factors such as emphysema and poor lung compliance, is a common occurrence that can significantly impact the surgical field. This condition often hampers the visibility of the surgical area, extends the duration of the operation, and amplifies the challenge of exposing the anatomical structures pertinent to the surgery. While adjusting the position of the DLT or applying suction may offer some relief, in the majority of cases, these interventions prove to be inadequate to achieve the desired level of lung collapse necessary for optimal surgical access [3].

During one‐lung ventilation (OLV), the process of lung collapse unfolds in a two‐stage sequence. Initially, on the side designated for surgery, the pleural cavity is opened, which alleviates the intrapleural pressure. This action, coupled with the lung's inherent elastic recoil, prompts the lung to retract and undergo a partial collapse. Subsequently, the gas within the lung is absorbed into the bloodstream, leading to a more pronounced collapse. It is this second phase that is considered the rate‐limiting factor in the collapse of the lung [4, 5]. Our objective, therefore, was to expedite the second step to enhance the quality and efficiency of lung collapse.

In the second phase of lung collapse, the propulsion and absorption of gas are predominantly governed by the concentration gradient between the pulmonary alveoli and the pulmonary vessels. The permeability of these pulmonary vessels also exerts a significant influence on the process. Several case reports have suggested a correlation between the inhalation of high concentrations of oxygen and the occurrence of postoperative atelectasis. Building on this insight, we hypothesized that varying the concentration of inhaled oxygen could potentially impact either the gas propulsion/absorption dynamics or the permeability of the pulmonary vessels, thereby accelerating the lung collapse process.

Some studies support the aforementioned hypothesis that maintaining a high inspired oxygen concentration during one‐lung ventilation (OLV) can lead to rapid collapse of the surgical lung, yet there is no clear recommendation for the optimal inspired oxygen concentration. Other research suggests that inhaling high concentrations of oxygen may not effectively improve the quality or speed of lung collapse [6]. On the contrary, inhaling high concentrations of oxygen during OLV could potentially increase the incidence of postoperative atelectasis. Most importantly, when high concentrations of oxygen enter the ischemic lung, they may trigger oxidative stress responses, leading to the production of a large number of superoxide compounds. When the lung is reperfused, this damage is amplified, severely affecting the patient's postoperative recovery [7, 8].

Hence, a pertinent question emerges: Does elevating the oxygen concentration influence the level of oxidative stress and augment the production of superoxides? In light of this, we embarked on a trial to ascertain whether high perioperative concentrations of inhaled oxygen could enhance lung collapse and to explore its potential impact on oxidative stress. We opted to investigate the effects of oxygen fractions (FiO2) at 70% and 100%.

In the trial, we documented the multidimensional scores of lung collapse in patients undergoing thoracoscopic surgery and compared the changes in oxidative stress indicators before and after surgery for each patient. The aim of the trial was to provide evidence for the selection of inspired oxygen concentrations during one‐lung ventilation surgery.

## 2 Methods

### 2.1 Study Design and Dates

**Figure 1:**
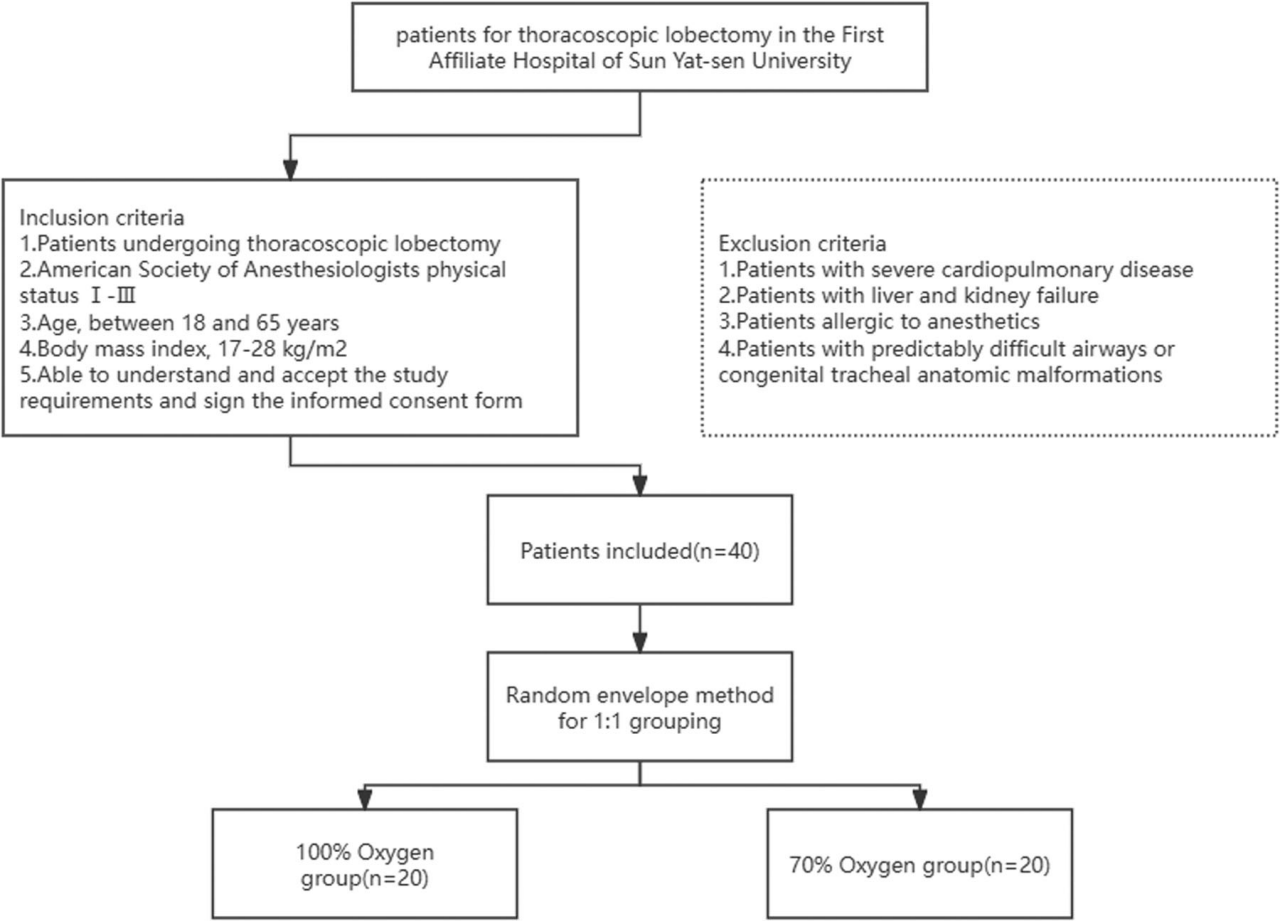
Flow chart for inclusion of patients.

This prospective, double‐blinded, randomized controlled trial was conducted at the First Affiliate Hospital, Sun Yat‐sen University. The study date was during nov. 2020 to Mar. 2021. Flow chart for inclusion of patients is shown in Figure 1.

### 2.2 Ethical Approval

Our study underwent rigorous review and received approval from the Ethics Committee of The First Affiliated Hospital, Sun Yat‐sen University, Guangzhou, China, chaired by Professor Yan Churong, on October 29, 2020. We are committed to sharing the research outcomes with the broader community through publication in peer‐reviewed scientific journals. The trial registration number is ChiCTR2100045495.

Furthermore, all participants provided their informed consent by signing the consent form, indicating their voluntary agreement to participate in the study.

### 2.3 Study Population

#### 2.3.1 Inclusion Criteria

1. Patients undergoing thoracoscopic lobectomy
2. American Society of Anesthesiologists physical status Ⅰ‐Ⅲ
3. Age, between 18 and 65 years
4. Body mass index, 17–28 kg/m^2^
5. Able to understand and accept the study requirements and sign the informed consent form

#### 2.3.2 Exclusion Criteria

1. Patients with severe cardiopulmonary disease
2. Patients with liver and kidney failure
3. Patients allergic to anesthetics
4. Patients with predictably difficult airways or congenital tracheal anatomic malformations

### 2.4 Randomization

Eligible patients were randomly assigned to one of two groups at a 1:1 ratio. The random envelope method was performed to achieve randomization. Briefly, in advance of study commencement, the researchers prepared a certain number of sealed envelopes that contained the information specific for each group. This information included different inhaled oxygen concentrations, the group number, and the times for collecting blood samples. The envelope accompanied the corresponding patient into the operating room. Then, the attending anesthetist checked the patient's qualifications. When the patient met the inclusion criteria, the anesthetist recorded the patient number on the outside of the envelope before opening the envelope. Then, the anesthetist opened the envelope and followed the instructions included.

The assessors were allowed to commence the experiment only after the attending anesthetist implemented the test procedure. The assessors (including the attending surgeon) and the patient were blinded to the group allocation until study completion. All researchers involved in the recruitment, consent, and outcome assessments were blinded to group allocations until study completion.

### 2.5 Sample Size Calculation

The main outcome measure of this study was the score of perioperative lung collapse. The smallest clinical difference in lung collapse scores was 2 points, and the standard deviation (SD) reported in previous trials has ranged from 0.8 to 1.3. We selected 1.0 as the SD for this trial. We set up two groups, distinguished by the FiO2: 70% oxygen and pure (100%) oxygen. Thus, we estimated the sample size with the following formula:

$${n=\varphi }^{2}\left(\sum S_{i}^{2}/g\right)/\left[\sum {\left({\bar{X}}_{i}-\bar{X}\right)}^{2}/\left(g-1\right)\right]$$

The N was 18, but considering data shedding, we determined that a sample size of 20 would be sufficient for each group.

### 2.6 Interventions

#### 2.6.1 Preoperative Preparation

The day before surgery, researchers conducted a preoperative visit with each patient, assessing their condition and explaining the study procedures. Following this, patients provided their informed consent by signing the consent form. The researchers then placed the group assignment envelope in the patient's medical record folder and discussed the satisfaction scoring criteria with the surgeons.

#### 2.6.2 Preparation after Entering the Operation Room

Upon patient identification, a peripheral IV line was established. The anesthetist verified the patient's eligibility, reviewed the envelope contents to determine the group assignment, and then the patient was taken to the operating room for baseline vital sign recording.

Before anesthesia, a radial artery catheter was inserted for blood sampling and gas analysis. A retrograde catheter was also placed in the internal jugular vein to obtain a sample from the jugular venous bulb for jugular venous bulb oxygen saturation (SjvO2) measurement. We calculated the CBF/CMRO2 ratio using the formula:

CBF/CMRO_2_ = 100/(1.34 × Hb × [SaO_2_ – SjvO_2_] + 0.003 × [PaO_2_ – PjvO_2_])

Additionally, we utilized the INVOS™ cerebral oximetry system to monitor regional brain oxygen saturation (rSO2) in both hemispheres of each patient.

#### 2.6.3 Anesthesia Induction and Management

Patients in each group received a target‐controlled infusion of propofol at 4.0 µg/mL, sufentanil at 0.5 µg/kg, and cisatracurium at 0.2 mg/kg for anesthesia induction. Once the drugs took effect, a DLT was inserted into the trachea under video laryngoscope guidance, positioning the lateral tube in the bronchus of the nonoperative lung side. The catheter was secured, and lung isolation for OLV was confirmed. Ventilation was maintained preoperatively with a tidal volume of 8 ml/kg, frequency of 12 breaths/min, and FiO2 as per group instructions.

Following intubation, anesthesia was sustained with propofol at 2.0 ng/mL and remifentanil at 4.0 ng/mL via target‐controlled infusion, along with half the induction dose of cisatracurium (~4–6 mg) administered every 60 min.2.6.4 Experimental interventions

Following anesthesia induction, the anesthetist adjusted the composition of the inhaled gases. The 40 enrolled patients were evenly divided into two experimental groups: one group received 70% oxygen during surgery (the 70% group), and the other received 100% oxygen (the 100% group). Ventilator settings were concealed to maintain assessor blindness. Both lungs were ventilated until the surgeon was ready to make the initial skin incision. At the time of inserting a trocar, the anesthetist turned the ventilation mode to OLV, and the inhaled gas composition was not changed until the operation was complete. Next, the surgeon inserted the thoracoscope into the chest through the trocar, and the assessors scored the quality of lung collapse, based on the thoracoscope image. Assessors will collaborate with the surgeon to conduct scoring procedures, which include palpating lung tissue to evaluate rebound and timing, or directing the thoracoscopic view towards the apex of the pleura to assess lung volume.

The scoring was conducted three times: at 0 min (baseline) and at 5 and 10 min after thoracoscope entered the thoracic cavity. Based on pilot observations in 10 cases, maximal lung collapse typically occurred within 8–12 min after pleural opening. We therefore selected assessment points at 50% (5 min) and near completion (10 min) of this critical window to capture the dynamic process. At each time point, the assessors also recorded the surgeon's satisfaction score.

**Figure 2:**
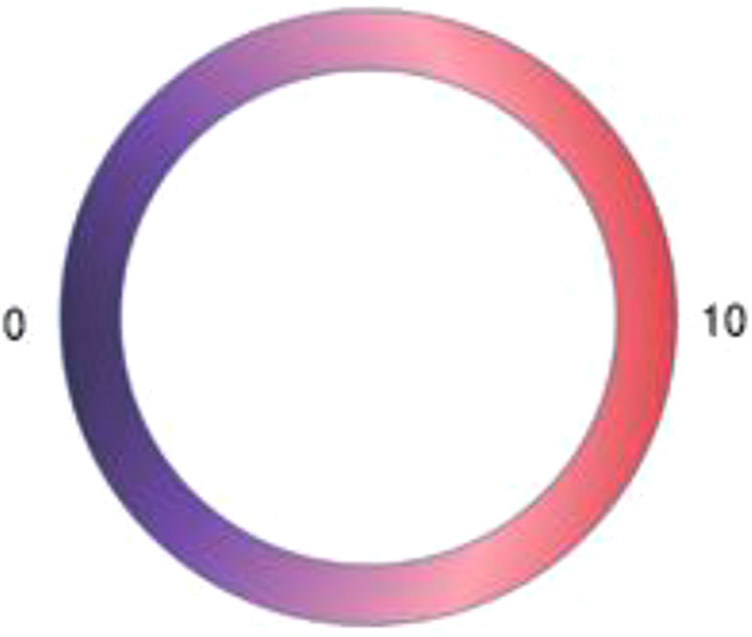
Color scale for lung color rating. Red is fully oxygenated (score = 10), and purple is ischemic (score = 0).

**Figure 3:**
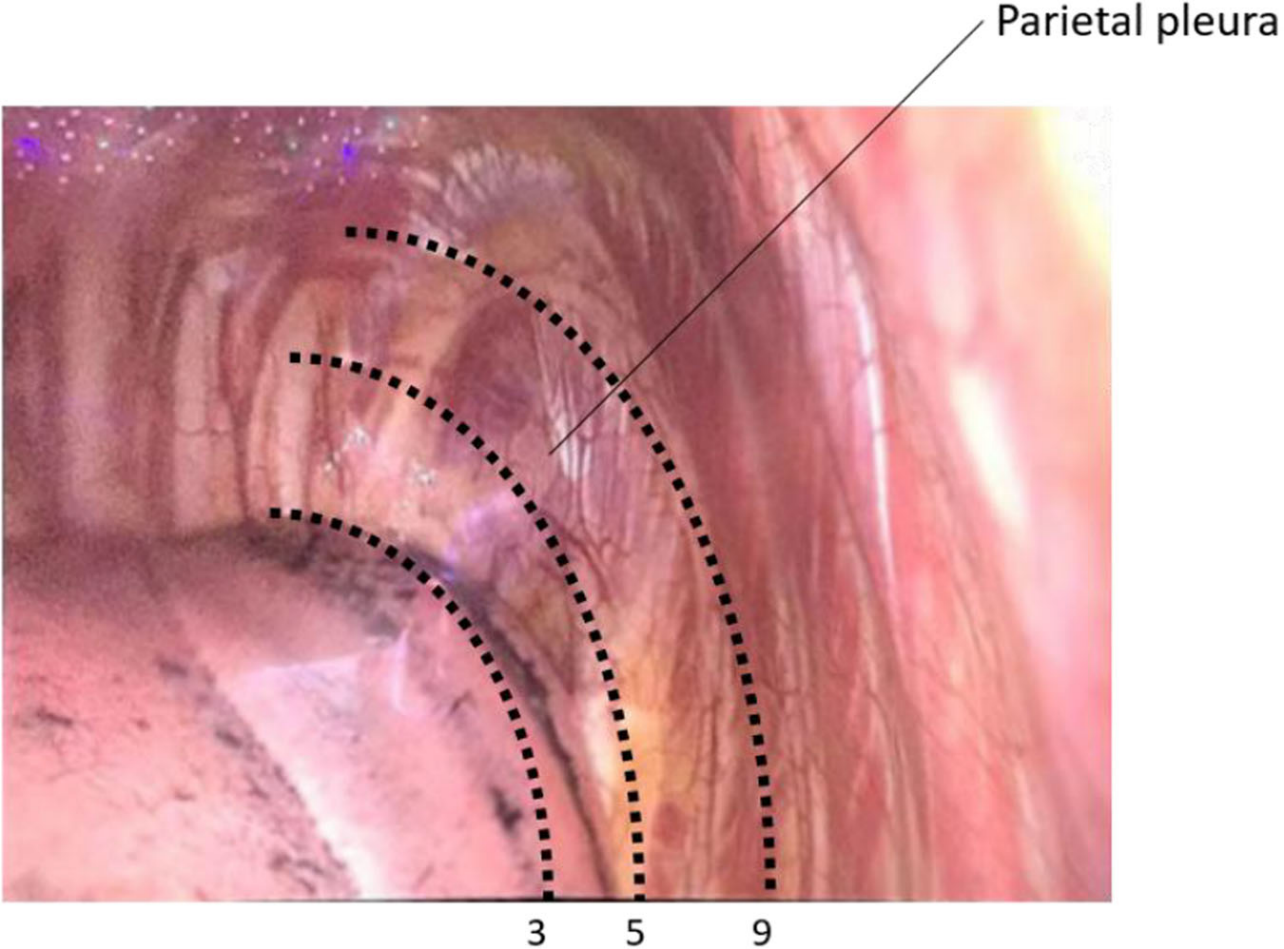
Reference for lung volume rating. The scale is 0 to 10, where 10 indicates that the lung fills the pleural space at the level of intercostal ribs 3 and 4. Dotted lines indicate the scores for alveoli collapse, when the lungs fill 90%, 50%, and 30% of the pleural space.

We modified the simple scoring system of lung collapse previously described by Li et al. [9] The scoring system for lung collapse included 3 different aspects: lung color, lung elasticity, and lung volume inside the thoracic cavity. The color scale ranged from 0 to 10: 10 for bright red and 0 for black lung tissue (Figure 2). Lung elasticity was quantified, based on the time it took the lung to rebound. The elasticity scale ranged from 0 to 10: 0 for an immediate, full rebound and 10 for no rebound. The assessors estimated the volume of lung tissue with video‐assisted thoracoscopy. They visually estimated how much (%) of the thoracic cavity was filled by the lung at the level of the third and fourth intercostal spaces (Figure 3). The maximum score was 10 points, for complete filling in each dimension, and a lung that filled 50% of the thoracic cavity was given 5 points. Thus, higher scores indicated slower speed of lung collapse.

In addition, 5‐mL blood samples were collected from the radial artery in anticoagulation tubes at 3 time points: before the operation, 30 min after the OLV, and the morning of the second postoperative day. Blood samples were immediately sent to the clinical laboratory to measure the concentrations of oxidative stress factors(include C‐reactive protein (CRP), superoxide dismutase (SOD), transferrin (Tf), serum ferritin (sFer), ischemia modified albumin (IMA)).

At the end of surgery, OLV was stopped at the time that the drainage tube was inserted into the thoracic cavity, and double lung ventilation was re‐established.

### 2.7 Follow Up

All patients were sent to the post anesthesia care unit (PACU) after the operation, and the endotracheal DLT was removed in the PACU. The anesthetist did not change the ventilator parameters before extubation. Adverse events were recorded in the PACU, including nausea, vomiting, stomach ache, and respiratory complications.

### 2.8 Database Locking

Data were recorded on a hardcopy (paper) case report form (CRF). All completed CRFs were placed into a locker, and access was limited solely to the clinicians involved in the study. After all the data were recorded, a re‐evaluation was performed to search for internal inconsistencies, range errors, or missing data. For each atypical, out‐of‐range, or missing datum, a query was sent to the researchers. After all the queries were resolved, the principal researchers, data collectors, and statistical analysts completed the final definition of the analysis population. Then, the database was used only for statistical analyses.

### 2.9 Statistical Analyses

All experimental data were analyzed using SPSS version 20.0. Quantitative data underwent the Shapiro‐Wilk test for normality. Data that were normally distributed are expressed as the Mean ± SD, while categorical variables are presented as frequencies. The serial changes in lung collapse scores and oxidative stress‐related factor levels were assessed using repeated measures analysis of variance. A p‐value of less than 0.05 was considered to indicate statistical significance.

## 3 Results

This study included 40 patients that underwent a thoracoscopic lobectomy. Baseline characteristics are presented in Table 1. There was no significant difference in age, sex, body weight, or other general characteristics between the two groups.

**Table 1:** Baseline characteristics of patients scheduled for thoracoscopic lobectomy.

Characteristics	70% oxygen (*n* = 20)	100% oxygen (*n* = 20)	t/χ² value	*p*‐value
Sex			0.11	0.744
Male	12	13		
Female	8	7		
Age (y)	49.5 ± 8.14, (45.69, 53.31)	53.1 ± 8.01, (49.35, 56.85)	1.41	0.167
BMI (kg/m2)	22.95 ± 3.26, (21.42,24.48)	24.02 ± 2.71, (22.74,25.30)	1.13	0.266
ASA‐PS			0.36	0.835
I	2	1		
II	17	18		
III	1	1		
Complications	12 (60%)	13 (65%)	0.11	0.744
Surgical site			0.44	0.507
Left lung	14 (70%)	12 (60%)		
Right lung	6 (30%)	8 (40%)		

*Note:* Values are the number (%) or mean ± SD, as indicated.

Abbreviations: ASA‐PS, American Society of Anesthesiologists‐Performance Status; BMI, body mass index.

**Figure 4:**
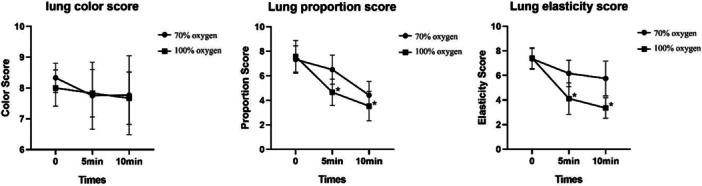
Alveoli collapse scores before (0 min) and after (5 and 10 min) entering the thoracic cavity. 70% oxygen (circles) is compared to 100% oxygen (squares). (*Left*) lung color; (*middle*) lung volume; (*right*) lung elasticity. Values are the mean ± SD. *means significantly different from the 70% group at the same time.

There was no significant difference in lung collapse scores between groups, upon entering the thoracic cavity (Table 2, Figure 4). At 5 and 10 min after entering the thoracic cavity, the lung volume and elasticity scores significantly decreased in each group, but they were significantly lower with 100% oxygen than with 70% oxygen (*p* < 0.05). However, the lung color scores were not significantly different between groups.

**Table 2:** Alveoli collapse scores before (0 min) and after entering the thoracic cavity.

Scores and measurement times	70% Oxygen (*n* = 20)	100% Oxygen (*n* = 20)	*p*‐value		
			Group	Time	Group × Time
Lung color score					
0 min	8.33 ± 0.47	8.00 ± 0.59	*p* = 0.585	*p* = 0.058	*p* = 0.472
5 min	7.75 ± 1.09	7.83 ± 0.77			
10 min	7.77 ± 1.28	7.67 ± 0.85			
Lung volume score					
0 min	7.33 ± 1.11	7.59 ± 1.29	*p* = 0.005	*p* < 0.001	*p* = 0.002
5 min	6.50 ± 1.19^a^	4.65 ± 1.08^a^, ^b^			
10 min	4.42 ± 1.11^a^	3.53 ± 1.19^a^, ^b^			
Lung elasticity score					
0 min	7.35 ± 0.85	7.41 ± 0.84	*p* < 0.001	*p* < 0.001	*p* < 0.001
5 min	6.17 ± 1.07^a^	4.12 ± 1.28^a^, ^b^			
10 min	5.75 ± 1.42^a^	3.35 ± 0.84^a^, ^b^			

*Note:* Values are the mean ± SD.

a significantly different from the 0 time measurement.

^b^ significantly different from the 70% group at the same time.

**Figure 5:**
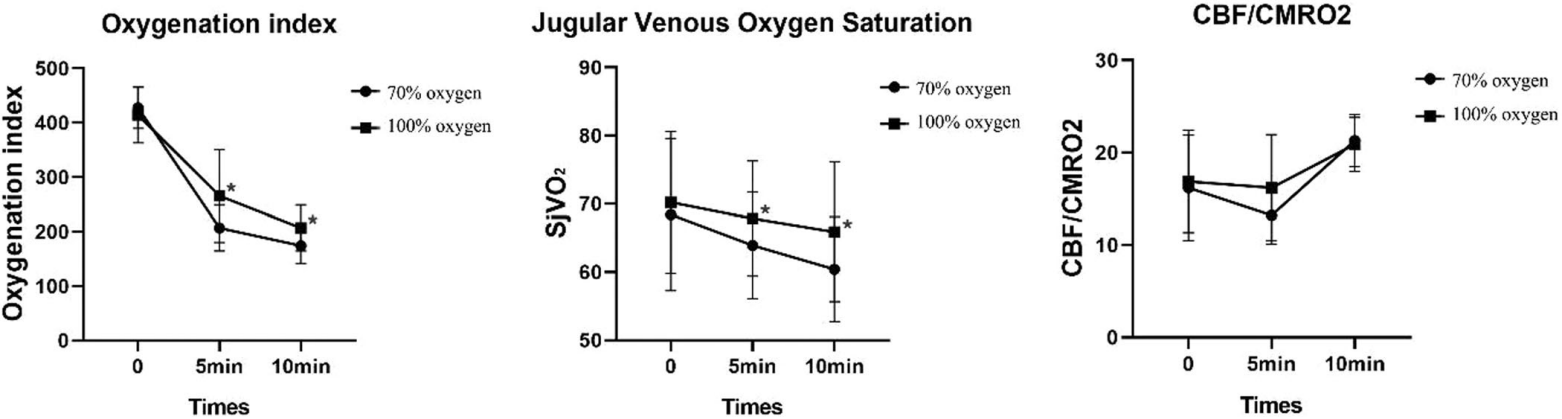
Lung oxygenation scores before (0 min) and after (5 and 10 min) entering the thoracic cavity. 70% oxygen (circles) is compared to100% oxygen (squares). (Left) Blood oxygenation index; (middle) Jugular vein oxygen saturation; (right) ratio of cerebral blood flow (CBF) to cerebral metabolic rate (CMRO2). Values are the mean ± SD. *means significantly different from the 70% group at the same time.

Before the operation, the groups showed no significant differences in oxygenation, including the oxygenation index, jugular sinus oxygen concentration, and transcutaneous cerebral oxygen saturation. After OLV was established, the indexes significantly decreased in both groups (Table 3, Figure 5), but the indexes were significantly lower with 70% oxygen than with 100% oxygen (*p* < 0.05).

**Table 3:** Arterial and cerebral oxygenation before and after a thoracoscopic lobectomy.

Oxygenation parameter	70% oxygen (*n* = 20)	100% oxygen (*n* = 20)	*p*		
			Group	Time	Group × Time
PaO_2_ (mmHg)					
0 min	320.50 ± 26.66	414.26 ± 51.24	*p* < 0.001	*p* < 0.001	*p* < 0.001
5 min	144.83 ± 29.62^a^	265.68 ± 85.39^a^, ^b^			
10 min	122.17 ± 22.83^a^	221.58 ± 74.43^a^, ^b^			
OI					
0 min	427.86 ± 38.08	414.26 ± 51.24	*p* = 0.008	*p* < 0.001	*p* = 0.007
5 min	206.90 ± 42.31^a^	265.68 ± 85.39^a^, ^b^			
10 min	174.52 ± 32.62^a^	206.90 ± 42.31^a^, ^b^			
Right rSO_2_ (%)					
0 min	70.25 ± 3.61	72.11 ± 3.11	*p* = 0.073	*p* < 0.001	*p* < 0.001
5 min	64.17 ± 4.52^a^	69.32 ± 3.08^a^, ^b^			
10 min	63.17 ± 4.43^a^	68.26 ± 2.67^a^, ^b^			
Left rSO_2_ (%)					
0 min	67.00 ± 3.39	69.47 ± 2.85	*p* = 0.532	*p* < 0.001	*p* < 0.001
5 min	62.42 ± 4.75^a^	66.68 ± 3.40^a^, ^b^			
10 min	61.50 ± 3.57^a^	64.47 ± 3.77^a^, ^b^			
SjVO_2_ (%)					
0 min	68.42 ± 11.15	70.21 ± 10.40	*p* = 0.678	*p* = 0.015	*p* = 0.032
5 min	63.92 ± 7.84^a^	67.84 ± 8.43^a^, ^b^			
10 min	60.42 ± 7.68^a^	65.89 ± 10.23^a^, ^b^			
CBF/CMRO_2_					
0 min	16.19 ± 5.73	16.87 ± 5.53	*p* = 0.235	*p* < 0.001	*p* = 0.192
5 min	13.22 ± 3.10^a^	16.20 ± 5.73			
10 min	21.29 ± 2.83^a^	20.88 ± 2.93^a^			

*Note:* Values are the mean ± SD.

Abbreviations: CBF/CMRO2, ratio of cerebral blood flow (CBF) to cerebral metabolic rate (CMRO2); PaO2, arterial partial oxygen pressure; rSO2, SjVO2, jugular venous bulb oxygen saturation.

a significantly different from the 0 time measurement.

^b^ significantly different from the 70% group at the same time.

**Figure 6:**
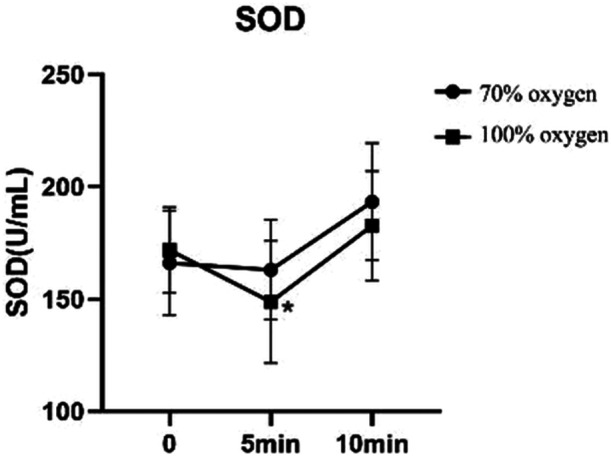
Changes in oxidative stress before (0 min) and after (5 and 10 min) the thoracoscopic lobectomy. Oxidative stress is indicated by the level of superoxide dismutase (SOD) measured in the blood. 70% oxygen (circles) is compared to100% oxygen (squares). Values are the mean ± SD. *means significantly different from the 70% group at the same time.

Before the operation, the groups were not significantly different in the oxidative stress indexes, based on the levels of SOD and CRP (Table 4, Figure 6). At 30 min after OLV was established, the SOD activity decreased significantly in both groups, but the activity was lower with 100% oxygen than with 70% oxygen (*p* < 0.05). On the second day after the operation, the SOD activity was significantly higher than at 30 min after OLV in both groups. In contrast, the CRP levels were not significantly different between groups before or at 30 min after OLV was established. However, CRP levels increased significantly on the second day after operation in both groups; and the increase was significantly higher in the 70% group than in the 100% group (*p* < 0.05).

**Table 4:** Oxidative stress indexes before and after a thoracoscopic lobectomy.

Stress indicator	70% oxygen (*n* = 20)	100% oxygen (*n* = 20)	*p*‐value		
			Group	Time	Group × Time
SOD (U/mL)					
0 min	166.08 ± 23.24	171.82 ± 19.15	*p* = 0.075	*p* < 0.001	*p* = 0.020
30 min	163.12 ± 22.20^a^	148.75 ± 27.19^a^, ^b^			
2nd po day	193.35 ± 26.03^a^	182.67 ± 24.32^a^			
CRP (mg/L)					
0 min	1.37 ± 1.87	1.35 ± 2.22	*p* = 0.091	*p* < 0.001	*p* = 0.120
30 min	1.39 ± 1.78	1.41 ± 3.47			
2nd po day	94.28 ± 23.14^a^	81.52 ± 27.76^a^, ^b^			
Tf (g/L)					
0 min	1.98 ± 0.31	1.93 ± 0.33	*p* = 0.643	*p* = 0.728	*p* = 0.632
30 min	1.89 ± 0.35	1.94 ± 0.36			
2nd po day	1.85 ± 0.33	1.94 ± 0.37			
sFer (µg/L)					
0 min	242.51 ± 122.82	237.51 ± 126.60	*p* = 0.996	*p* = 0.692	*p* = 0.906
30 min	235.23 ± 122.69	234.29 ± 115.65			
2nd po day	257.62 ± 109.38	255.82 ± 118.62			
IMA (U/mL)					
0 min	79.43 ± 2.43	80.74 ± 1.93	*p* = 0.853	*p* = 0.225	*p* = 0.069
30 min	81.16 ± 2.79	80.46 ± 2.49			
2nd po day	81.69 ± 3.81	81.36 ± 2.71			

*Note:* Values are the mean ± SD.

Abbreviations: CRP, C‐reactive protein; IMA, ischemia‐modified al‐†umin; po, postoperative; SOD, superoxide dismutase; Tf, transferrin; sFer, serum ferritin.

a significantly different from the 0 time measurement.

^b^ significantly different from the 70% group at the same time.

There was no significant difference between groups in the levels of Tf, sFer, or IMA (*p* > 0.05).

There was no significant difference in safety outcomes between the two groups of patients (*p* > 0.05) shown in Table 5.

**Table 5:** Safety outcomes after a thoracoscopic lobectomy.

Safety outcomes	70% oxygen (*n* = 20)	100% oxygen (*n* = 20)	χ² value	*p*‐value
Hypoxemia	2	2	0	1.000
Atelectasis	2	4	0.78	0.376
Pneumonia	1	0	1.03	0.311
Intraoperative hemorrhage	0	1	1.03	0.311

## 4 Discussion

With advancements in thoracic surgery, thoracoscopic pneumonectomy has increasingly supplanted open thoracic procedures. In recent years, the detection rate of pulmonary tumors in China has risen significantly [10, 11]. However, from the perspective of thoracic anesthetists, there is a need to enhance operational processes to minimize complications and improve efficiency.

The OLV is a critical component of thoracic surgery, serving as the foundation for successful operations. Anesthetists must ensure complete lung isolation to provide optimal exposure of the surgical field while simultaneously maintaining adequate oxygen supply and minimizing the risk of oxidative stress injuries during OLV [12, 13].

This study demonstrated that varying concentrations of inhaled oxygen influence both the speed and quality of lung collapse [14]. This study demonstrated that varying concentrations of inhaled oxygen influence both the speed and quality of lung collapse. We compared the effects of 70% oxygen against pure oxygen, assessing lung volume, color, and elasticity. Lung volume is a key factor affecting the surgical process, as the dissolution of gas in the lungs impacts thoracic cavity volume. Additionally, the loss of alveolar gas during dissolution reduces lung lobe elasticity. Observations from video‐assisted thoracoscopy indicate that lung tissue can be easily compressed [15]. To accurately evaluate the gas dissolution rate, we assessed lung volume and elasticity scores. Furthermore, since oxygen is the primary gas exchanged during intrapulmonary gas exchange, we also included lung color evaluation as an indirect measure of oxygen dissolution.

Our initial findings revealed no significant differences between the groups. However, shortly after initiating OLV, both groups experienced a notable decrease in lung volume and elasticity, indicating a reduction in pulmonary gas due to OLV. Notably, these reductions were more pronounced in the pure oxygen group compared to the 70% oxygen group, under identical ventilator settings and similar lung compliance conditions. This suggests that pure oxygen dissolves into the bloodstream more quickly than 70% oxygen within the alveoli.

The gas dissolution into the bloodstream is the rate‐determining step for lung collapse. With equivalent thoracic elasticity, the rate of lung collapse is directly proportional to the rate of gas dissolution, which is in turn linked to the concentration of the gas. A higher gas concentration creates a greater pressure differential between the alveoli and blood vessels, facilitating faster dissolution. Consequently, the 100% oxygen group demonstrated superior lung elasticity and volume scores.

Upon thoracoscope entry into the chest cavity, changes in lung color score were minimal, with little variation between groups. This could be attributed to the ongoing oxygen supply from the pulmonary vessels, which were still intact during the initial phase of cavity entry.

The oxygenation data revealed that the use of pure oxygen during surgery significantly enhanced blood oxygen levels. Notably, indices related to brain oxygenation also increased. This result aligns with previous research demonstrating the benefits of high oxygen concentrations for brain oxygen uptake [16]. In addition, we may infer that patients with cerebral ischemia might benefit from pure oxygen inhalation.

We then explored the optimal inhaled oxygen concentration to minimize oxidative stress injury while maintaining effective oxygenation during one‐lung ventilation (OLV). Blood samples were collected to assess levels of CRP, SOD, Tf, sFer, and IMA, which reflect three distinct aspects of oxidative stress. CRP, associated with inflammation and tissue trauma, indicates tissue injury when elevated due to its role in the acute‐phase response. SOD, which converts superoxide free radicals into less harmful peroxides, suggests free‐radical accumulation when levels are low, protecting tissues and cells from damage during hypoxia and reperfusion. Tf, sFer, and IMA are indicators of blood loss‐related injuries; their altered levels signal inadequate perfusion [17].

The oxidative stress markers yielded contrasting results, with significant differences in CRP and SOD levels between the groups, yet no variance in ischemia‐related indicators. This data can be interpreted with certain experimental presuppositions. Initially, during OLV, we presumed that the blood supply to the lung tissue remained undisturbed before resection and that only a portion of the target tissue had its blood vessels ligated. Under these assumptions, it's plausible that there were no differences in ischemia markers between the groups.

In the pure oxygen group, intraoperative hyperoxia might have rapidly generated a surge of free radicals, leading to a substantial consumption of the body's stored SOD due to high blood free‐radical levels. However, by the following day, SOD activity had risen markedly in both groups, with no significant intergroup differences. We speculate that this increase coincided with the restoration of physiological respiratory status and improved oxygen supply in both groups.

There are still some limitations in this experiment. Firstly, we could have employed a wider range of methods to assess lung collapse, particularly by utilizing ultrasound examinations. This approach would provide an objective quantification of lung collapse, independent of the subjective judgment of surgeons. However, during the experimental design phase, some researchers were not proficient in performing ultrasound lung scans, leading us to ultimately abandon this method [18, 19]. Additionally, in the selection of oxygen concentrations, we only compared 70% oxygen with pure oxygen. This decision was based on our preexperimental findings that the incidence of intraoperative hypoxemia increased when the concentration dropped below 70%. Perhaps in future studies, we could adopt a more nuanced gradient of concentrations to validate our experiment, thereby offering more suggestions for selecting the appropriate oxygen concentration for patients undergoing one‐lung ventilation.

Additionally the lack of formal inter‐rater reliability testing for the lung collapse scoring system may raise concerns about measurement consistency. Although we implemented a rigorous single‐evaluator‐with‐consultation protocol to maintain scoring uniformity, future studies would benefit from pretrial rater training and statistical reliability testing.

In summary, high/pure inhaled oxygen can enhance lung collapse speed and surgical visibility in thoracic surgery. Our study indicates that increasing oxygen concentration during OLV can quickly improve systemic oxygenation and benefits cerebral oxygen uptake. Although pure oxygen can induce oxidative stress, its effects appear transient. Thus, we recommend higher inhaled oxygen concentrations to optimize lung collapse and oxygenation during OLV, with vigilant monitoring for oxidative stress.

## Author Contributions

**Dizhou Zhao:** conceptualization, data curation, methodology, project administration, writing – original draft, writing – review and editing. **Chujun Wu:** conceptualization, data curation, formal analysis, investigation, resources. **Haoshuai Zhu:** investigation, resources. **Jun Lin:** project administration. **Wangzhi Zhang:** methodology. **Jieyu Fang:** investigation, methodology, resources, supervision, validation.

## Conflicts of Interest

The authors declare no conflicts of interest.

## Declarations

All authors have read and approved the final version of the manuscript Pro. Fang had full access to all of the data in this study and takes complete responsibility for the integrity of the data and the accuracy of the data analysis.

All research funding was self‐funded and was primarily used to pay for nutritional supplements for subjects participating in the trial.

Pro. Fang affirms that this manuscript is an honest, accurate, and transparent account of the study being reported; that no important aspects of the study have been omitted; and that any discrepancies from the study as planned (and, if relevant, registered) have been explained.

All experimental data are publicly shared, and you can contact Pro. Fang by email to obtain the data.

## 1 Transparency Statement

The lead author Jieyu Fang affirms that this manuscript is an honest, accurate, and transparent account of the study being reported; that no important aspects of the study have been omitted; and that any discrepancies from the study as planned (and, if relevant, registered) have been explained.

## Data Availability

All experimental data are publicly shared, and you can contact Pro. Fang by email to obtain the data.
